# Characterization of the Blood Bacterial Microbiota in Lowland Tapirs (*Tapirus terrestris*), a Vulnerable Species in Brazil

**DOI:** 10.3390/microorganisms12112270

**Published:** 2024-11-08

**Authors:** Anna Claudia Baumel Mongruel, Emília Patrícia Medici, Rosangela Zacarias Machado, Keith Clay, Marcos Rogério André

**Affiliations:** 1 Vector-Borne Bioagents Laboratory (VBBL), Faculdade de Ciências Agrárias e Veterinárias (FCAV), Universidade Estadual Paulista “Júlio de Mesquita Filho” (UNESP), Jaboticabal 14884-900, São Paulo, Brazil; anna.mongruel@unesp.br (A.C.B.M.); rz.machado@unesp.br (R.Z.M.); 2 Lowland Tapir Conservation Initiative (LTCI), Institute for Ecological Research (IPÊ), Campo Grande 79046-150, Mato Grosso do Sul, Brazil; medici@ipe.org.br; 3 Escola Superior de Conservação Ambiental e Sustentabilidade (ESCAS/IPÊ), Nazaré Paulista 12960-000, São Paulo, Brazil; 4 Tapir Specialist Group (TSG), International Union for Conservation of Nature (IUCN SSC), Campo Grande 79046-150, Mato Grosso do Sul, Brazil; 5 Department of Ecology and Evolutionary Biology, School of Science and Engineering, Tulane University, New Orleans, LA 70118, USA; clay@tulane.edu

**Keywords:** Brazilian tapir, NGS, wild animals, microbiome

## Abstract

Microbiome studies targeting hypervariable regions of the 16S rRNA gene are suitable for understanding interactions between animals and their associated bacteria. While many studies focus on the gut microbiome, assessments of blood microbiota remain scarce despite the prevalence of blood-borne pathogens in vertebrates. This study aimed to investigate the bacterial community in blood samples from 79 living and 7 road-killed lowland tapirs (*Tapirus terrestris*), a vulnerable species, sampled in two biomes in midwestern Brazil: Pantanal and Cerrado. Animals were categorized by condition (living or road-killed), sex, age, and biome. V3–V4 16S rRNA fragments were obtained from 86 blood samples and 4 negative controls. After filtering contaminants, 13,742,198 sequences representing 2146 ASVs were analyzed. Alpha diversity significantly differed by condition, while beta diversity differed by condition, site, and age (adults vs. sub-adults). For living animals (79/86 samples), alpha diversity showed no significant differences, but beta diversity differed by age. Different vector-borne bacterial pathogens, including *Anaplasmataceae*, *Bartonella*, and *Borrelia* spp., were detected. Additionally, evidence of transient translocation of microbial communities from other body regions to the bloodstream was observed. Amplification of bacterial 16S rRNA from blood samples of wild *T. terrestris* provided novel information about the diversity of blood-borne microbiota of lowland tapirs, members of a poorly studied mammalian family. Next-generation sequencing proved to be a valuable tool for screening potential vector-borne pathogens in this host.

## 1. Introduction

Research focusing on interactions between microorganisms and animal hosts has increased significantly over the last decades, including many studies investigating the host’s microbiome. Microbiomes are assemblages of microbial species with strong levels of interaction among them and determined by the environment within which the microbiome occurs [1]. The relationship between the microbiome and its host is described as one of the most complex and intimate biological communities known [2].

Studies on microbiomes are increasing due, in part, to the growing access to new technologies such as next-generation sequencing (NGS) and computational tools for bioinformatic analyses [1]. The use of NGS is particularly advantageous given that it can identify non-cultivable species, especially when there have been no prior studies of the bacterial community present in a certain biological system [3]. Both whole-genome sequencing (WGS) and sequencing of informative regions from the 16S ribosomal (rRNA) gene can be used for microbial identification. In fact, rRNA genes are considered universal and conservative targets useful for bacteria discovery and identification [4].

In non-domesticated wild animals, microbiome studies are often linked to efforts focused on species conservation. The study of oral and/or gut microbiota of critically endangered species, such as amphibians [5], rhinoceros [6], and birds [7,8], has been used for elucidating how dietary and environmental changes may affect microbiome composition and host health. Moreover, surveillance of pathogens in these animals is fundamental for identifying or preventing the emergence of infectious diseases in wild animals as well as in humans and domestic animals [3]. Although many studies have focused on the gut microbiome of vertebrates [5,6,7,8,9,10], microbiome assessments of blood samples are scarce [11,12], despite the known prevalence of diverse blood-borne pathogens.

The genus *Tapirus* (Family Tapiridae, Order Perissodactyla) comprises four extant species, with additional species known only from the fossil record [13]. While one species (*Tapirus indicus*, Desmarest, 1819) occurs in Asia, the other three species occur only in Central and South America (*Tapirus pinchaque* [Roulin, 1829], *Tapirus terrestris* [Linnaeus, 1758], and *Tapirus bairdii* [Gill, 1865]) [14,15]. Currently, *T. indicus*, *T. pinchaque*, and *T. bairdii* are considered endangered, while *T. terrestris* is considered vulnerable to extinction [16]. The lowland tapir (*T. terrestris*) can be found in multiple countries in South America, including Argentina, Bolivia, Brazil, Colombia, Ecuador, French Guiana, Guyana, Peru, Suriname, and Venezuela [17]. Despite the fact that this species can be found in four distinct biomes across Brazil (Pantanal, Cerrado, Amazon, and Atlantic Forest), it is classified as vulnerable throughout the country based on its risk of extinction due primarily to human actions such as hunting, domestic livestock production, wild fires, and road-kills [18].

Although no studies have previously investigated the blood microbiome of lowland tapirs using bacterial 16S rRNA amplification through NGS, applying this approach could provide insights into the bacterial communities present in the bloodstream of this vulnerable species and its ecology. The overarching goal of the present study was to investigate the bacterial communities present in blood samples from wild tapirs sampled in the Pantanal and Cerrado biomes in midwestern Brazil by amplification and sequencing of the V3–V4 hypervariable region of the bacterial 16S rRNA. Our results add to our understanding of the blood microbiota of wild mammals and vulnerable species, and therefore potentially contribute to their conservation.

## 2. Methods

### 2.1. Sampling

**Figure 1:**
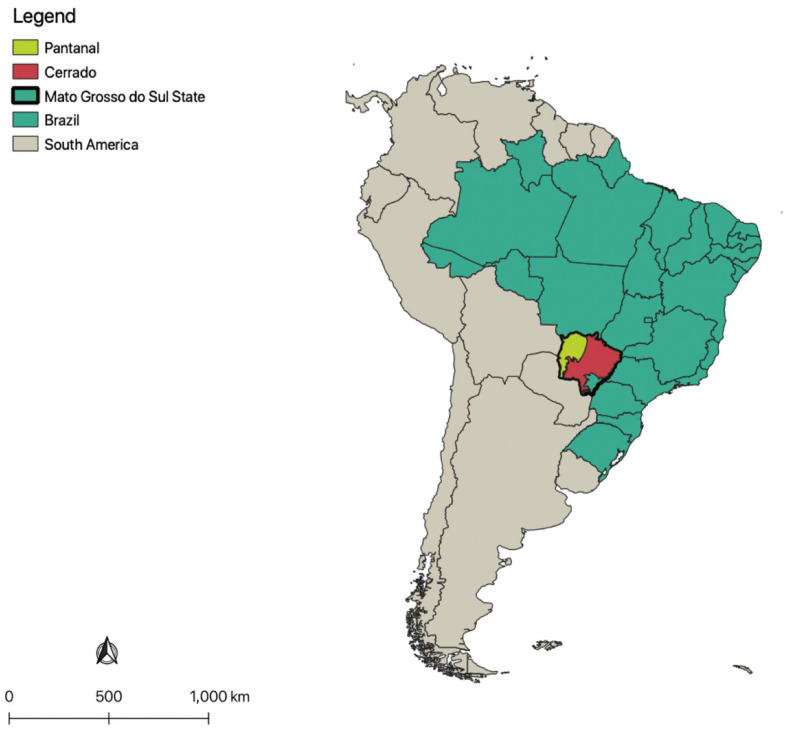
Map of Brazil (divided by states and highlighted in South America) showing the location of Pantanal and Cerrado biomes within Mato Grosso do Sul State.

From 2013 to 2018, blood samples from wild *T. terrestris* were collected from 94 living and 8 road-killed individuals, totaling 126 samples (some living animals were sampled more than once at different times). Out of these, 78 blood samples were collected from 61 tapirs in the Pantanal wetlands and 40 samples were collected from 33 tapirs from the Cerrado biome. Additionally, 8 road-killed tapirs from the Cerrado biome were sampled during necropsy procedures, for a total of 41 individuals from this biome. Animals were identified based on sex (males and females) and estimated ages (adults: >48 months old; sub-adults: 18–47 months old; juveniles: <18 months old). Both biomes and all sample sites are located in Mato Grosso do Sul State, central-western Brazil (Figure 1).

Blood sampling was performed during tapir anesthesia for the installation of GPS collars by professionals from the Lowland Tapir Conservation Initiative (LTCI), part of the Institute for Ecological Research (LTCI/INCAB—IPÊ). Blood samples (up to 2 mL each) were maintained in a −20 °C freezer until further analysis. All procedures were approved by the Ethics Committee for Animal Experimentation of FCAV/UNESP (Faculty of Agricultural and Veterinary Sciences of the São Paulo State University) under protocol number 4558/20. The “Instituto Chico Mendes de Conservação da Biodiversidade (ICMBIO)” provided the required annual permits for the capture and immobilization of tapirs and collection of biological samples (SISBIO# 14,603).

All protocols for the capture, anesthesia, handling, and sampling of tapirs were reviewed and approved by the Veterinary Advisors of the Association of Zoos and Aquariums (AZA) Tapir Taxon Advisory Group (TAG), and the Veterinary Committee of the IUCN SSC Tapir Specialist Group (TSG). Tapir blood DNA samples from the present study were registered in the Brazilian National System for Management of Genetic Heritage and Associated Traditional Knowledge (Sistema Nacional de Gestão do Patrimônio Genético e do Conhecimento Tradicional Associado—SISGEN) under register number AE4CC0C.

### 2.2. DNA Extraction

DNA extraction procedures were performed at the Vector-Borne Bioagents Laboratory, UNESP, Jaboticabal (Brazil), using a commercial kit (DNeasy Blood and Tissue Kit Mini Spin, Qiagen, Hilden, Germany) and following manufacturers’ instructions. The kit was opened and used exclusively for processing the samples from the present study. In order to monitor potential contaminants, DNA extraction procedures were performed in six different rounds, and two negative controls were included in each round (totaling 12 negative controls). One negative control from each round contained only reagents from the extraction kit (without any blood sample). The other negative control of each round comprised ultra-purified sterilized water (Nuclease-Free Water, Promega^®^, Madison, WI, USA) in addition to extraction kit reagents. In all rounds, extraction was performed by one person using examination gloves and a mask. Prior to each extraction round, the working bench was cleaned with three different solutions in the following order: enzymatic detergent (Zymedet Gold, Prolink, Guapiaçu, Brazil), 70% ethanol, and 10% sodium hypochlorite (bleach). Additionally, the entire room was exposed to UV light for 15 min. DNA concentration of each sample was measured using a Qubit 4 Fluorometer (Thermo Fisher Scientific, Waltham, MA, USA) and samples with a minimum concentration of 12 ng of DNA/sample (average volume of DNA per sample of 38 μL) were selected for sequencing.

### 2.3. Sequencing

The DNA samples were shipped from Brazil to New Orleans (LA, USA) (U.S. FWS permit #MAPER5230668). Library preparation of the 16S rRNA V3–V4 regions were performed at the LSU Translational Genomics Core (New Orleans, LA, USA) using Illumina^TM^ MiSeq equipment (San Diego, CA, USA). The primer sequences used to amplify an approximately 450 pb fragment of the bacterial 16S rRNA gene were Bakt_314F 5′-CCTACGGGNGGCWGCAG-3′ and Bakt_805R 3′-GACTACHVGGGTATCTAATCC-5′ [19]. Illumina sequencing adapters and dual-index barcodes were added using the full complement of Nextera XT indices (Illumina, Inc., San Diego, CA, USA), following the instructions proposed by the Illumina protocol [20]. The pooled final DNA library was sequenced using paired 300 bp reads that overlapped at their ends, generating high-quality, full-length reads for the targeted V3 and V4 regions. Run outputs typically exceeded 20 million reads and, considering there were 96 indexed samples, more than 100,000 reads per sample were produced on average, which is commonly recognized as adequate for metagenomic surveys [20].

### 2.4. Bioinformatics Data Processing

Demultiplexed raw fastq files were processed using QIIME2 (Quantitative Insights into Microbial Ecology) [21]. Sequence trimming, primer removal, denoising, and singleton removals were performed using the Divisive Amplicon Denoising Algorithm 2 (DADA2) implemented in QIIME2. The classifier was trained using the Greengenes2 2022.10 99% database [22] to classify sequences. A table with the frequency of each ASV per sample was created and exported to RStudio 4.2.3 software for analysis using the microDecon package [23]. The package identified and removed contaminant sequences, based on sequences and their frequencies found in negative controls and cross-contamination. Negative controls were also removed from any further analyses after this step. Additionally, the plugin “filtering” on QIIME2 was based on previous reports of contaminants present in low biomass microbiome studies [24]. Data compilation and graphics were performed using Microsoft Excel 2016 software.

### 2.5. Diversity and Statistical Analyses

Diversity measures and statistical significance were calculated using the plugin “diversity” on QIIME2 [21]. Diversity analyses were divided into two sections: 1. all samples combined (living and road-killed animals); and 2. living animals only. For both analyses, rarefaction curves were constructed in order to avoid any bias caused by variations in sequencing depth. For alpha diversity, both richness and evenness were calculated using Shannon’s metrics and statistical significance was computed using Kruskal–Wallis (significance was considered when *p*-value < 0.05). For beta diversity, the Principal Coordinate Analysis (PCoA) was used to construct an ordination plot, together with Weighted Unifrac, for the calculation of dissimilarity of the microbial communities. Statistical analysis was performed using the Permutational Multivariate Analysis of Variance test (PERMANOVA) to calculate if distances were significant among groups (significance was considered when *p*-value < 0.05). Community analyses, using non-metric multidimensional scaling (NMDS) [25], were also performed using the package “vegan” v2.6-2 [26] and package “phyloseq” v.1.4 [27] on R software.

## 3. Results

### 3.1. Obtained Sequences

From 89 tapir blood DNA samples and 12 negative controls subjected to NGS, fragments from the V3–V4 hypervariable region of the bacterial 16S rRNA were successfully obtained from 86 samples (86/89; 96.62%) and 4 (4/12; 33.33%) negative controls.

Regarding the negative controls that generated sequences, two were from reagents and water controls and the remaining two from reagent controls only. Of the samples that were successfully sequenced, 46 were obtained from the Pantanal biome and 40 were obtained from the Cerrado biome. Table 1 describes the total number of samples obtained for each category (site, sex, and age) from all samples combined. In addition, samples from living animals only are described separately.

### 3.2. Primer Removal, Denoising, and Filtering of the Obtained Sequences

Primers were successfully removed from all sequence reads. Forward and reverse sequences were trimmed in lengths of 260 bp and 199 bp, respectively, and an overlap of 15 bp was obtained after construction of the contigs. A total of 31,769 amplicon sequence variants (ASVs) were identified, with a total of 32,555,385 sequences obtained and a mean value per sample (MSD) of 361,727. After identifying and filtering mitochondrial and unidentified sequences from the database, the total number of ASVs dropped to 5532, with a total of 30,582,559 sequences and an MSD of 339,806. The total number of ASVs then dropped to 5380 with 17,446,143 total sequences and an MSD of 202,862. For the removal of additional contaminants, the dataset was initially filtered based on a list of taxa reported as contaminants in microbiome studies of low biomass samples. Moreover, taxa found in the present study were also manually reviewed for the identification of potential contaminants. The list of removed ASVs by microDecon and all keywords included in the Qiime2 filtering command are presented in Supplementary File S2 and Supplementary File S3, respectively. After filtering out contaminants, the final total number of ASVs obtained was 2146, with a total of 13,742,198 sequences and an MSD of 159,793.

### 3.3. ASV Diversity

**Figure 2:**
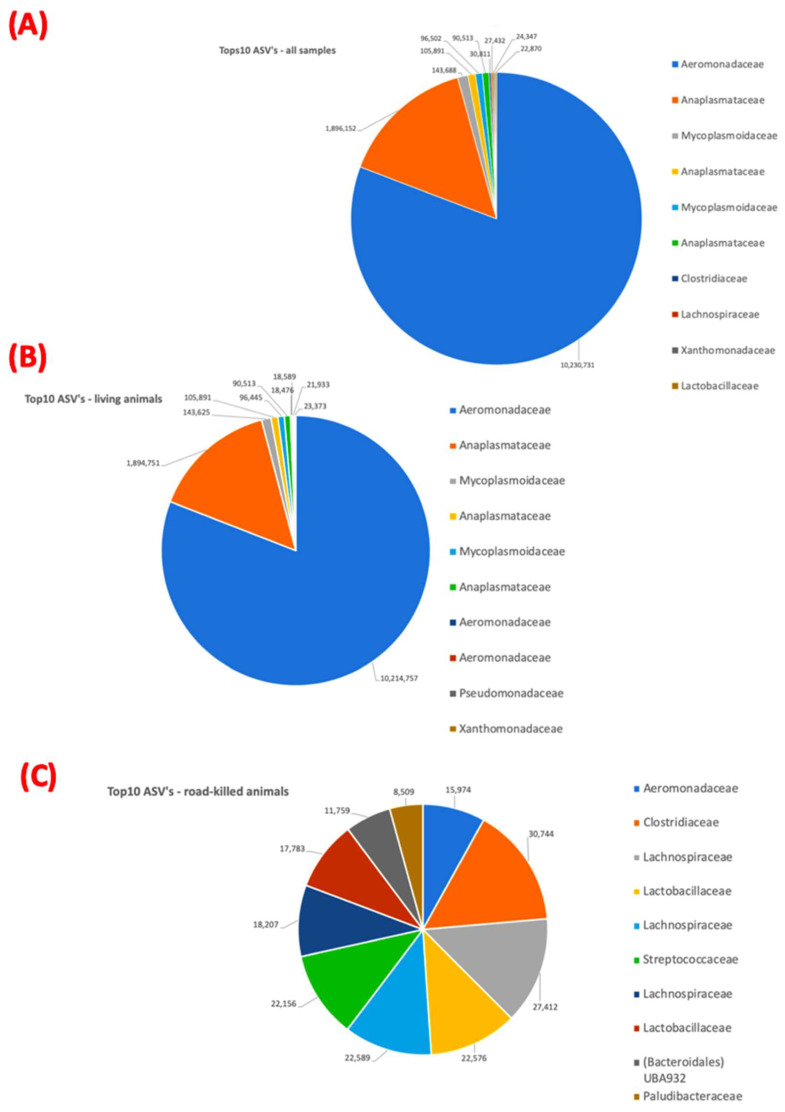
Representation of the top 10 ASVs from all samples (living and road-killed) (**A**), samples of living animals (**B**), and road-killed animals (**C**).

For a better visualization of the most frequent taxa found herein, the 10 most frequent taxa (top 10) were highlighted and compared to the remaining taxa found in each group (all samples, living animals, and road-killed animals). Pie charts were used to represent the frequency distribution of the top 10 taxa (Figure 2).

**Figure 3:**
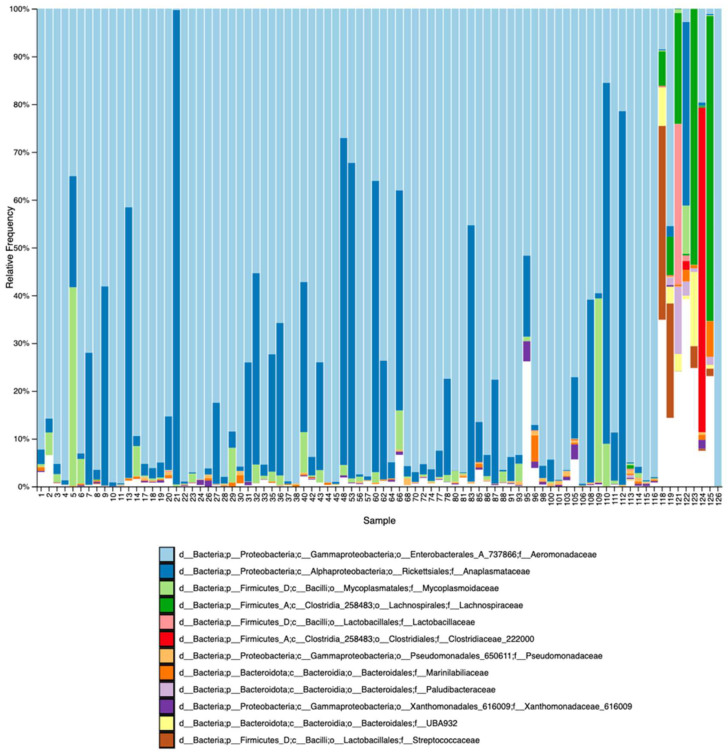
Taxonomic bar plot demonstrating the relative frequency of the 12 most frequent ASVs identified in taxa in analyzed samples (living and road-killed tapirs). ASVs are identified until rank 5 (Family). Samples 118 to 125 represent road-killed animals.

A taxonomic bar plot (Figure 3) was also generated to illustrate the relative frequency of the 10 most frequent ASVs detected in all samples after the identification and removal of contaminant sequences. Due to limited taxonomic identification provided by the sequence length obtained in the present study, sequences were identified until taxonomic rank number 5 (Family). A table containing the identification of each sample number regarding sex, site, age, and condition is available in Supplementary File S1.

Phyla Proteobacteria, Firmicutes, Bacterioidota (formerly Bacterioidetes), Actinomycetota (formerly Actinobacteria), and Spirochaetota (formerly Spirochaetes) were the dominant taxa present in our analyses. The Aeromonadaceae and Anaplasmataceae families were the two most frequent bacterial groups found when all samples (from living and road-killed animals) were analyzed together. The top 10 most frequent ASVs represented 92% of all ASVs found in all samples. Aeromonadaceae and Anaplasmataceae were also the two most frequent taxa groups found in samples from living animals only. The top 10 ASVs represented 95% of all ASVs found in these samples. The top 10 from all samples and living animal samples only were largely similar. By contrast, Clostridiaceae was the most frequent taxonomic group found in samples from road-killed animals. Unlike for living animals, the top 10 ASVs in road-killed samples represented 45% of all ASVs found. The composition of the top 10 ASVs from road-killed animal samples was different from the top 10 ASVs found when all samples were analyzed together and when samples from living animals were analyzed separately. When compared with samples from living animals, the second most frequent taxa group (Anaplasmataceae) did not appear in the top 10 in road-killed animal samples. The most frequent group for living animal samples (Aeromonadaceae) was the eighth most frequent group for road-killed animal samples.

### 3.4. Diversity Analysis of All Samples—Alpha Diversity

**Figure 4:**
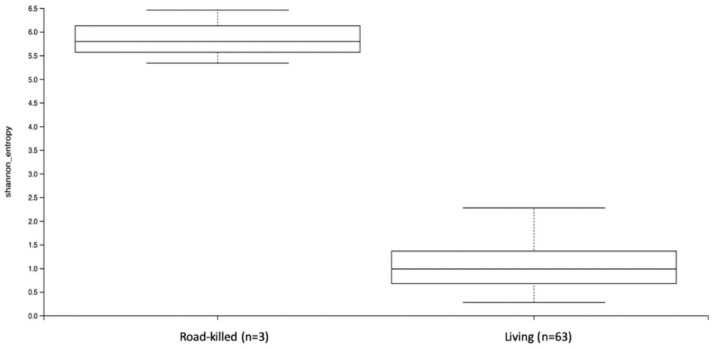
Shannon’s metric bar plots comparing the obtained values of alpha diversity between blood samples of living and road-killed animals. The only statistically significant variable was condition (living or road-killed animals) (*p*-value = 0.0036).

The maximum sample depth obtained for this analysis was 428,598. The rarefaction value of 88,183 was chosen for alpha diversity measurements once it retained 42.35% (5,820,078) of the total frequency of ASVs in 76.74% (*n* = 66) of the samples at the specified sample depth. Shannon’s entropy metric was chosen to evaluate both species’ richness and evenness. From all variables analyzed (site, sex, age, and condition), the only statistically significant variable was condition (living or road-killed animals) (*p*-value = 0.0036) (Supplementary File S4). The bar plot for this result is shown in Figure 4. The variables of sex, age, and site were not statistically significant for Shannon’s metric. Based on this result, the number of different bacteria, and how evenly distributed these bacteria are in terms of abundance within the samples, were significantly different between samples from living and road-killed animals.

### 3.5. Diversity Analysis of All Samples—Beta Diversity

**Figure 5:**
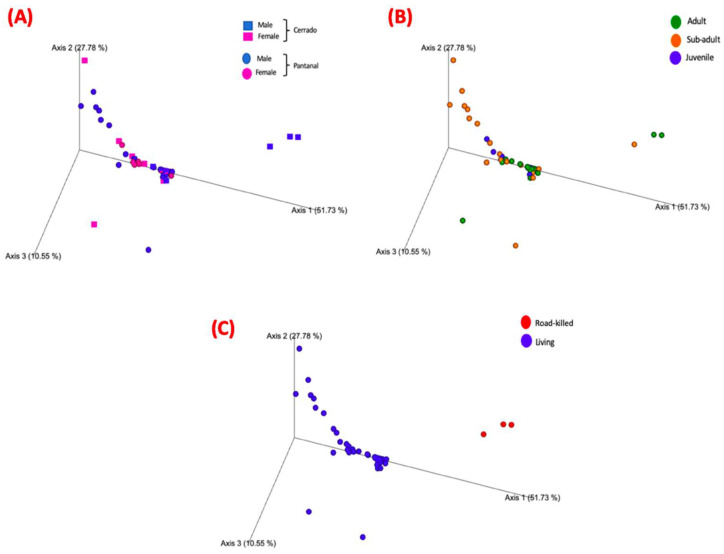
Weighted Unifrac distances plot for the distribution of the analyzed samples. (**A**) Samples from adult tapirs are highlighted in green, samples from sub-adult tapirs are highlighted in orange, and samples from juvenile tapirs are highlighted in blue. (**B**) Samples from females are highlighted in pink, and samples from males are highlighted in blue. Samples from Pantanal biome are represented by spheres, and samples from Cerrado biome are represented by squares. (**C**) Samples from living animals are highlighted in blue while samples from dead (road-killed) animals are highlighted in red.

The same rarefaction point (88,183) was used for beta diversity analysis. PCoA was used together with Weighted Unifrac for the calculation of distances for the communities in beta diversity analysis. Figure 5 shows the Weighted Unifrac distances plot for the distribution of the samples analyzed.

**Figure 6:**
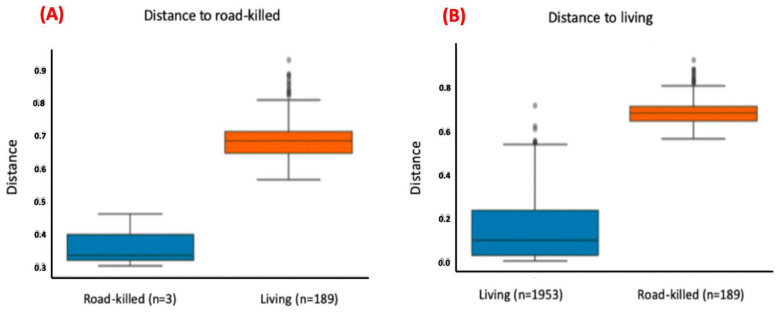
Bar plots comparing the distances between bacterial communities found in blood samples of living and dead (road-killed) animals. Plot (**A**) computes the distances of communities found in blood samples within living animals and between living and road-killed animals. Plot (**B**) computes the distances of communities found in blood samples within road-killed animals and between road-killed animals and living animals. Bacterial communities present in samples from living and road-killed animals showed statistically significant differences (*p*-value = 0.001).

**Figure 7:**
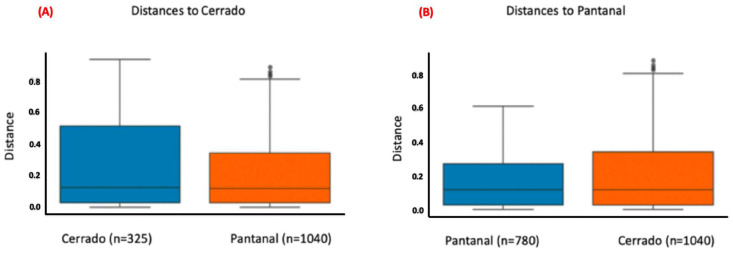
Bar plots comparing the distances between bacterial communities found in tapir blood samples from Cerrado and Pantanal. Plot (**A**) computes the distances of communities found in tapir blood samples within Cerrado animals and between Cerrado and Pantanal animals. Plot (**B**) computes the distances of communities found in tapir blood samples within Pantanal animals and between tapirs from Pantanal and Cerrado. Bacterial communities present in samples from Cerrado and Pantanal biomes showed statistically significant differences (*p*-value = 0.03).

**Figure 8:**
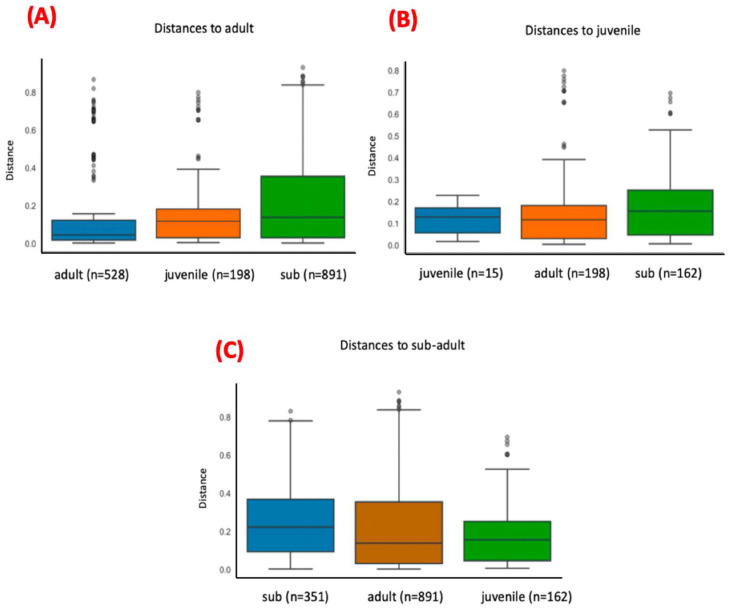
Bar plots comparing the distances between bacterial communities found in blood samples from adult, sub-adult, and juvenile tapirs. Plot (**A**) computes the distances of communities found in blood samples within adult tapirs and between adults and sub-adults, and between adults and juveniles. Plot (**B**) computes the distances of communities found in blood samples within juvenile tapirs and between juveniles and adults, and between juveniles and sub-adults. Plot (**C**) computes the distances of communities found in blood samples within sub-adult tapirs and between sub-adults and adults, and between sub-adults and juveniles. Bacterial communities present in samples from adult and sub-adult animals showed statistically significant differences (*p*-value = 0.007).

For this analysis, the variables condition (*p*-value = 0.001), site (*p*-value = 0.03), and age (adult x sub-adult; *p*-value = 0.007) showed statistically significant differences (Figure 6, Figure 7 and Figure 8; Supplementary File S4), but the variable sex did not. The pairwise comparisons between adults and juveniles or sub-adults and juveniles also did not show statistically significant differences.

### 3.6. Analysis of Living Animals’ Blood Samples

As described above, the composition of bacterial communities presented in blood samples from road-killed animals appeared to be significantly different from those obtained from blood samples from living animals. It is likely that this difference results from decomposition and bacterial proliferation in road-killed animals. For this reason, sequences from road-killed animals were filtered out and additional analysis were performed with samples from only living animals to test if the other variables may influence the alpha and beta diversity metric. A total of 13,276,936 sequences were retrieved from 79 samples, representing 1257 ASVs. The MSD value obtained was 168,062 with minimum and maximum sample depth values of 7270 and 428,598, respectively.

### 3.7. Diversity Analysis of Living Animals’ Blood Samples—Alpha Diversity

The maximum number of sequences per sample obtained in this analysis was 428,598. The rarefaction value of 86,212 was chosen for alpha diversity measurements once it retained 41.56% (5,517,568) of the total frequency of ASVs in 81.01% (*n* = 64) of the samples at the specified sample depth. Shannon’s entropy metric was chosen to evaluate both species’ richness and evenness in this analysis. None of the variables analyzed (site, sex, and age) showed statistically significant differences.

### 3.8. Diversity Analysis of Living Animals’ Blood Samples—Beta Diversity

**Figure 9:**
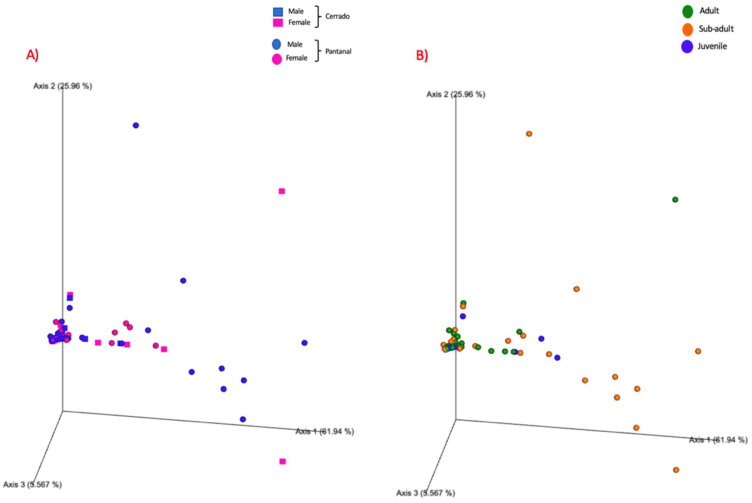
Weighted Unifrac distances plot for the distribution of the bacterial community found in living animals’ blood samples. (**A**) Samples from Pantanal are represented by squares whereas samples from Cerrado are represented by circles. Samples from females are highlighted in pink whereas samples from males are highlighted in blue. (**B**) Samples from adult animals are highlighted in green, samples from sub-adult animals are highlighted in orange, and samples from juvenile animals are highlighted in blue.

**Figure 10:**
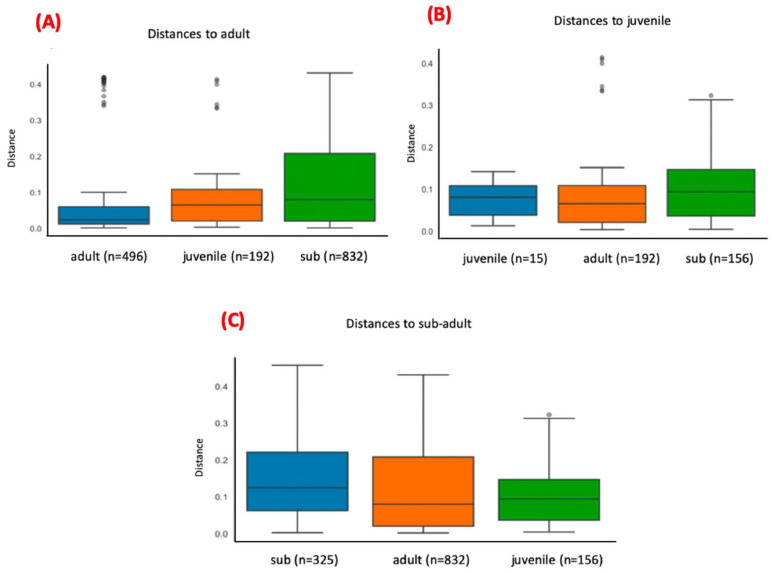
Bar plots comparing the distances between bacterial communities found in blood samples from adults, sub-adults, and juveniles in living animals’ samples. Plot (**A**) computes the distances of communities found in blood samples within adult animals and between adults and sub-adults, and between adults and juveniles. Plot (**B**) computes the distances of communities found in blood samples within juvenile animals and between juveniles and adults, and between juveniles and sub-adults. Plot (**C**) computes the distances of communities found in blood samples within sub-adult animals and between sub-adults and adults, and between sub-adults and juveniles. Bacterial communities present in samples from living adult and sub-adult animals showed statistically significant differences (*p*-value = 0.001).

The same rarefaction point (86,212) was used for beta diversity analysis. PCoA was used as part of the ordination approach together with Weighted Unifrac for the calculation of distances of the communities in beta diversity analysis. Figure 9 shows the Weighted Unifrac distances plot for the distribution of the analyzed samples. For this analysis, only age (adult vs. sub-adult) presented statistically significant differences (*p* = 0.01) (Figure 10, Supplementary File S4). Other variables (sex or site) did not show any statistically significant differences.

### 3.9. Diversity Analysis of Living Animals’ Blood Samples—NMDS

**Figure 11:**
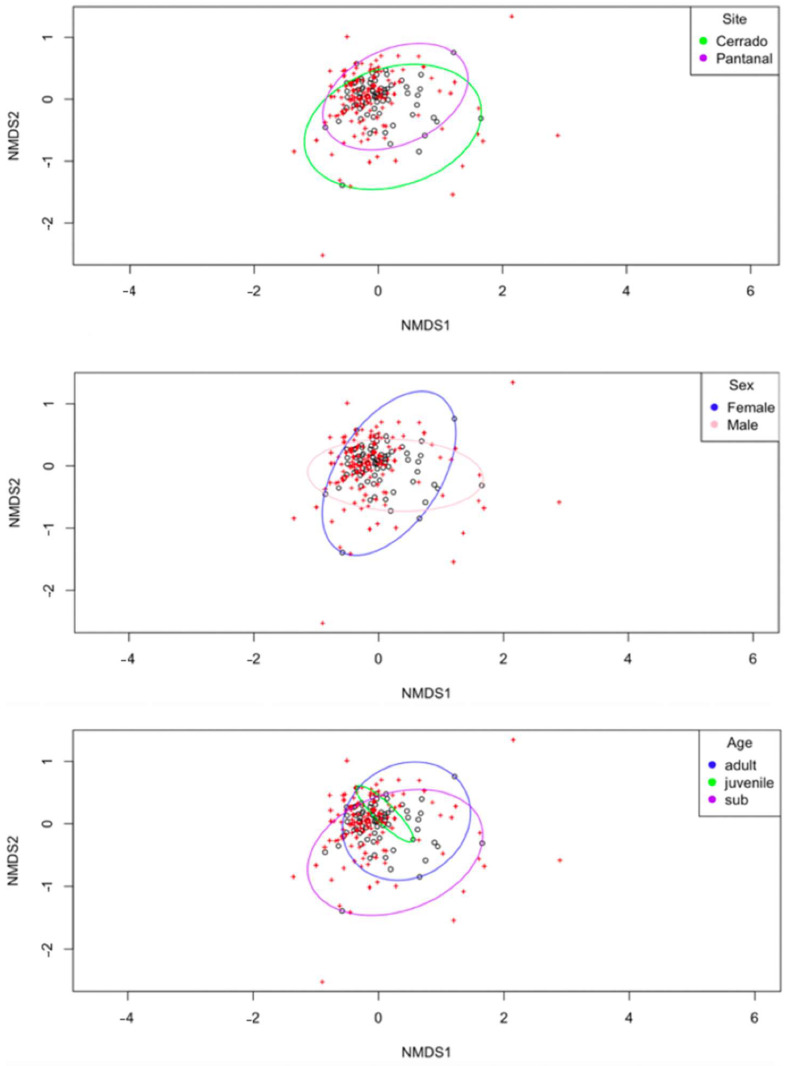
NMDS analysis performed using Bray–Curtis dissimilarity and Presence/Absence (PA) standardization on RStudio 4.2.3 software. The ellipses generated based on each variable (site, sex, and age) seemed to collapse, indicating no clear differences among the groups.

An NMDS analysis was performed to analyze the distribution of points and community composition based on the relative frequencies of ASVs in each sample and the variables using Bray–Curtis dissimilarity and Presence/Absence (PA) standardization, in the “vegan” package, and the ordiellipse function on R software (Figure 11). All generated ellipses were largely overlapping, demonstrating that there is little correlation among variables and beta diversity. The stress metric obtained from the analysis was 0.1600915, which is within a satisfactory rate (<0.2), and the best solution was repeated 1 time in 27 tries.

## 4. Discussion

### 4.1. Composition of Main Taxa Found in the Present Analysis

We report here results of the first investigation of the blood bacterial microbiota of lowland tapirs, a species considered vulnerable to extinction in Brazil. For many years, blood was believed to be a sterile environment and the presence of microorganisms in the blood was an indication of infection. However, with the advancement of molecular and sequencing techniques, the occurrence of a “healthy blood microbiome” has been widely discussed [28,29]. While some studies describe the existence of human blood microbiota [29,30,31], a recent investigation reported the absence of conclusive evidence of a common blood microbiome in humans. Instead, the study suggested that bacterial DNA in blood may be linked to transient translocation of microbial communities from other body sites into the bloodstream [32]. Dysbiosis is defined as a change in the composition of commensal communities compared to the composition found in healthy individuals. The source of dysbiosis is an interruption of the homeostasis which might be produced by inflammation, infection, metabolic diseases, or external interventions (e.g., use of antibiotics) [33]. Indeed, while circulating bacterial DNA can be found in blood, the bacteria must be cultivable to be considered viable [34]. Moreover, it is suggested that the intestinal barrier plays a role in limiting the bacterial groups that can enter the bloodstream under healthy conditions [35].

Previous studies have reported that Proteobacteria, Actinomycetota, Firmicutes, and Bacterioidota phyla are the most prevalent in the human blood microbiome [29]. In our study using lowland tapirs’ blood samples, Proteobacteria, Firmicutes, Bacterioidota, Actinomycetota, along with Spirochaetota, were also the dominant phyla found. Similar results were obtained in the investigation of blood mononuclear cells microbiome in goats [35] and whole blood microbiome in cows [36]. Most of the Proteobacteria found in our study were represented by Gammaproteobacteria and Alphaproteobacteria. Proteobacteria are one of the most abundant bacterial phyla and are considered to cause intestinal and extraintestinal diseases [37]. In a microbiome analysis of *Amblyomma scalpturatum* and *Amblyomma ovale* ticks collected from *T. terrestris* in Peru, members of the *Acinetobacter* genus, a member of Proteobacteria, were the most abundant taxa found [38].

Firmicutes and Bacterioidota include bacteria linked to the digestion of fiber and polysaccharides, respectively. One previous study that compared microbiome gut composition of captive and wild bharals (*Pseudois nayaur*) reported a higher frequency of Firmicutes in wild animals compared to captive animals, but a lower frequency of Bacterioidota, which was linked to differences in dietary habits between these two feeding categories [39]. Moreover, an increase in dietary fiber composition has also been linked to an increased Firmicutes:Bacterioidota ratio in monogastric animals [40]. Wild lowland tapirs’ diet is composed of large portions of plant fibers and smaller proportions of fruits [41], which suggests that a higher prevalence of Firmicutes compared to Bacterioidota was expected in the animals sampled in the present study.

The former phylum Tenecurites, which was exclusively represented by Mycoplasmadoiceae (formerly Mycoplasmataceae), is now classified as within the Firmicutes. Hemoplasmas (hemotropic *Mycoplasma* spp.) are small pleomorphic bacteria from the Mycoplasmadoiceae family which attach to the surface of erythrocytes of different mammalian hosts and may be a causative agent of anemia [42]. Hemoplasmas have been reported in a variety of wild animal hosts across different taxonomic groups in Brazil, including capybaras (*Hydrochoerus hydrochaeris*) [43], anteaters (*Tamandua tetradactyla*) [44], jaguars (*Panthera onca*) [45], procyonids such as coatis (*Nasua nasua*) [46], crab-eating foxes (*Procyon cancrivorus*) [47], and aquatic mammals such as pink river dolphins (*Inia* spp.) [48] and fur-seals (*Arctocephalus australis*) [49]. In fact, the occurrence of two different *Candidatus* species of hemoplasmas was previously reported from the same studied tapir population used in the present project [50]. Another major group found was Actinomycetota, which comprise a large number of bacteria related to the pathogens of animals and humans, but also occur as gut commensals [51]. This phylum also comprises a large number of bacteria from the human skin microbiota [29]. In the present study, Actinomycetota was mostly composed of Coriobacteriia and Actinomycetia, which have been described as commensal gut microorganisms [52]. Further, Actinomycetota was also described as part of the blood microbiome in healthy human adults [30].

To our knowledge, the present work presents the first microbiome analysis of blood samples from the genus *Tapirus* based on the amplification of the V3–V4 region of the bacterial 16S rRNA. An earlier study of the fecal microbiome of *T. bairdii* using similar techniques has been recently published, in which the fecal microbiome of Baird’s tapirs was dominated by Firmicutes, Bacteroidota, Proteobacteria, Kiritimatiellaeota, and Spirochaetota [53]. Here, the blood bacterial microbiome of lowland tapirs was dominated by phylum Proteobacteria, Firmicutes, Bacterioidota, Actinomycetota, and Spirochaetota. Considering the composition of Proteobacteria, *T. bairdii*’s fecal microbiome revealed a vast majority of Gammaproteobacteria, albeit with a small portion of Alphaproteobacteria [53]. In the present study, the dominant phylum was Gammaproteobacteria, mostly represented by Aeromonaceae, followed by Alphaproteobacteria, which was represented by Anaplasmataceae. These differences are expected considering the different types of biological samples used (feces vs. blood). In our study, we worked with blood samples, which allowed the detection of blood-borne bacteria, such as those from family Anaplasmataceae. In general, gut-related bacteria are expected to be found in higher prevalence in fecal samples.

Interestingly, the sequence representing the most frequent ASV observed in living animals’ samples exhibited a 100% identity with *Aeromonas hydrophila* (GenBank access numbers: KT998822, KX756709, OR726313) in BLASTn analysis. This species belongs to the mesophilic group of *Aeromonas*, which exhibit motility and have been associated with infection in humans. Moreover, it can thrive in a wide range of pH and temperature conditions [54]. Considering that *Aeromonas* species are frequently isolated from aquatic environments, the possibility of sample contamination cannot be discarded. However, *A. hydrophila* is also described as a ubiquitous fish pathogen and an opportunistic pathogen in humans [54], with reports of infection in cattle [55] and sheep [56], suggesting its potential to affect different animal hosts, including tapirs.

### 4.2. Sequence Recovery

Initially, sequencing recovered 32,555,385 sequences, 31,769 ASVs, and MSD of 361,726 for a dataset of 86 samples and 4 negative controls. After identification and removal of contaminant sequences, these counts dropped to a total frequency of 13,742,198 sequences, 2146 ASVs, and MSD of 159,793 for the dataset of 86 samples, leading to drops of 57.79%, 93.25%, and 55.83% for total frequency, number of ASVs and MSD, respectively. In comparison, a study using blood samples from camels (*Camelus dromedarius*) from Sudan obtained drops of 17.5%, 37.1%, and 12.9% for total frequency, number of ASVs and MSD, respectively, after the removal of contaminant sequences [57]. These contrasts may be explained by the larger number of analyzed samples, inclusion of different animal categories (living and road-killed) in our study, and because we accounted for sequences obtained from negative controls in our study, allowing the subsequent elimination of higher rates of contaminants.

### 4.3. Detection of Vector-Borne Related Taxa in Tapirs’ Blood Samples

Different taxa related to known vector-borne pathogens were found in tapir blood in the present study. Bacteria from the Anaplasmataceae family made up a large portion of the taxa found in all samples and in living animal samples—indeed, comprising the second most frequent ASV. A similar NGS-approach study using camels’ blood samples also reported a high frequency of Anaplasmataceae [57]. Recognized and novel genotypes of Anaplasmataceae have been molecularly detected and reported in different groups of wild vertebrates from Brazil, such as wild canids and felids [58,59,60], coatis (*Nasua nasua*) [61], anteaters and sloths [62], deer [63,64,65], and birds [66,67,68,69]. Considering such diversity of Anaplasmataceae in wildlife from Brazil, microbiome studies targeting variable regions of the 16S rRNA can act as important tools to reveal the diversity of members from this family and how they differ among host species [70]. Sequences from the family Bartonellaceae were also found in the present study. Recently, a *Bartonella henselae*-like genotype was described in the same studied population included in the present study based on amplification of the *nuoG* and *ribC* genes [71]. *Bartonella henselae* is the primary causative agent of Cat Scratch Disease in humans [72].

Sequences from the family Spirochaetota were found in the present study. Spirochaetota comprises helical bacteria that can be found as free-living or host-associated bacteria. Certain pathogenic strains pose threats to humans and animals, such as species from the genus *Borrelia* [73]. When one Spirochaetota sequence found in two Pantanal tapirs was compared with sequences deposited in the GenBank database using the BLASTn software (https://blast.ncbi.nlm.nih.gov/, accessed on 26 June 2024), there was 100% identity with sequences of *Borrelia theileri* previously detected in ruminants (GenBank accession numbers: MN621894, MN621893, MN619805). *Borrelia* spirochetes are causative of multiple diseases in humans and animals and can be transmitted by hard (Ixodidae) and soft (Argasidae) ticks [74]. One of the best-known pathogenic representatives from the genus is *Borrelia burgdorferi*, the causative agent of Lyme disease, a multisystemic zoonosis [75], but occurrence of this pathogen remains uncertain in Brazil [76]. The *Borrelia* 16S rRNA sequence found here showed strong resemblance to sequences from *B. theileri*, the causative of bovine borreliosis and part of the Relapsing Fever Group (RFG) *Borrelia* that are transmitted by hard ticks, specifically *Rhipicephalus* spp. [77,78], which have previously been reported infesting tapirs in the Pantanal and Cerrado regions [79]. In Brazil, *B. theileri* has been described in *Rhipicephalus microplus* [77,80,81] and cattle [82,83]. Recently, this species was reported in the same studied population of tapirs from the Pantanal biome using qPCR and conventional PCR assays [84].

### 4.4. Comparative Analysis of the Blood Microbiota of Living and Road-Killed Tapirs

The necrobiome of clotted blood samples from road-killed tapirs was also performed. The organic matter available after the death of a host continues the interaction between the host and its microbiota, making host decomposition the last stage of symbiosis [85]. The composition of the bacterial communities between living and dead animals was very different, and this was expected due to the decomposition process. Regarding diversity analyses for all samples, the condition (living or dead) variable was statistically significant for both the alpha diversity metrics used here. For beta diversity, variables condition, site, and age (adults vs. sub-adults) were significant. Considering the marked differences in composition between living and road-killed samples, these results might be explained by the fact that, after rarefaction, samples of road-killed animals were represented by two adults and one sub-adult from Cerrado.

The top 10 ASVs from live and dead tapir blood samples exhibited clearly different compositions. For live animals, Aeromonaceae and Anaplasmataceae families were dominant members of the top 10 components. By contrast, dead animals presented a more diversified group of ASVs, with Clostridiaceae and Lachnospiraceae families representing the most prevalent taxa. In the initial phases of decomposition, depletion of oxygen allows the propagation of anaerobic bacteria, such as Clostridiaceae, derived from the gastrointestinal tract [86]. Lachnospiraceae is a phylogenetically and morphologically diverse family that comprises bacteria of the core of the gut microbiome [87]. Higher abundances of Lachnospiraceae were also reported in early stages of decomposition in the human gut microbiome [88]. Similarly, Lactobacillaceae, which was the third most present ASV in road-killed samples, represent a varied group of lactic-acid producing bacteria found in the gastrointestinal tract of humans and animals [89], and can also proliferate during early stages of decomposition [90].

### 4.5. Bacterial Diversity in Tapirs’ Blood Microbiome

When samples of living animals were analyzed separately, Shannon’s entropy metric (alpha diversity) did not present statistical significance. Although the NMDS plot generated ellipses that largely overlapped for all variables, beta diversity metrics exhibited differences for age (adult vs. sub-adult). Considering that samples of road-killed animals were not included in the analysis, the blood microbiota composition of living adults differed from that of sub-adults, which were estimated to be between 18 and 47 months old. Reproductive maturity is reported to occur between 17 and 48 months old in lowland tapirs [91], and calves stay with their mothers until approximately 12–18 months old [92]. The influence of puberty and hormonal changes on immunological traits is reported in humans [93]. However, more studies are necessary to understand if hormonal, behavior, or dietary factors associated with this age group (puberty) may influence the blood microbiome in lowland tapirs.

### 4.6. Contaminants in the Present Analysis

Reporting potential contaminants is an important contribution for metagenomic studies [94]. Blood samples are considered low microbial biomass samples and are easily contaminated by contaminant DNA and/or cross-contamination during the processing of samples [95]. Indeed, even though recommended precautions to avoid contamination were taken during the DNA extraction process [3], the number of ASVs dropped by 38.79% (from 5532 to 2146) after the steps of identification and filtering out of contaminants. It is worth noting that samples from the present project were initially collected to perform a health assessment of wild tapirs where specific sampling procedures for microbiome analysis [95] were not taken, which might have allowed field contamination. Moreover, well-to-well contamination during the sequencing processes has also been reported [96]. Even though a large number of ASVs were eliminated after the filtering steps, we recognize that some contaminant sequences might have remained, including those present in high frequencies. ASVs identified as contaminants and removed in the present study were reported (Supplementary File S2).

In the present study, we found different microbial taxa that may be related to the microbiota of distinct anatomical sites but also to arthropod-borne infections. Here, amplification and NGS of bacterial 16S rRNA from blood samples of wild *T. terrestris* provided information about the diversity of bacterial agents found in lowland tapirs and potential vector-borne pathogens for this species.

## 5. Conclusions

The phyla Proteobacteria, Firmicutes, Bacterioidota, Actinomycetota, and Spirochaetota were the dominant taxa present in the analysis of the microbiome of living and road-killed lowland tapirs using amplification and NGS of a fragment of approximately 400 pb from the V3–V4 regions of the bacterial 16S rRNA. Frequent phyla found herein matched with taxa described as commensal for the blood and gut microbiome in other mammal and *Tapirus* species.

The composition of the most frequent ASVs between samples from living and road-killed animals differed extensively as expected due to the process of decomposition in road-killed animals. For living tapirs, diversity analyses suggest that the dominant community members are likely shared among tapirs across age and biomes (Pantanal and Cerrado). Age appears to be a significant variable for beta diversity of living tapirs.

Sequences from different vector-borne bacteria were found in the present study, mostly represented by Anaplasmataceae and Mycoplasmadoiceae families. The hemoparasite-related taxa found herein matched with findings from other studies conducted using blood samples from the same tapir population and qPCR and PCR assays, indicating that the NGS assay was a quick and useful tool for the molecular detection of these agents in lowland tapir blood.

## Figures and Tables

**Table 1:** Description of samples successfully sequenced with percentages from each category. Samples were divided into two groups: all samples (living and road-killed animals) and samples from living animals only.

Category		All Samples (Living and Road-Killed Animals)	Samples from Living Animals Only
Site	Pantanal	46 (46/86; 53.49%)	46 (46/79; 58.22%)
	Cerrado	40 (40/86; 46.51%)	33 (33/79; 41.77%)
Sex	Female	39 (39/86; 45.34%)	36 (36/79; 45.57%)
	Male	47 (47/86; 54.66%)	43 (43/79; 54.43%)
Age	Adult	44 (44/86; 51.16%)	40 (40/79; 50.63%)
	Sub-adult	33 (33/86; 38.37%)	31 (31/79; 39.24%)
	Juvenile	09 (9/86; 10.46%)	08 (8/79; 10.12%)
Total		86	79

## Data Availability

The original contributions presented in the study are included in the article/Supplementary Materials, further inquiries can be directed to the corresponding author. Reads from the present project are deposited in the Sequence Read Archive (SRA) (https://www.ncbi.nlm.nih.gov/sra, accessed on 4 November 2024) upon access PRJNA1176187.

## Supplementary Materials

The following supporting information can be downloaded at: https://www.mdpi.com/article/10.3390/microorganisms12112270/s1, File S1: Table containing the identification of each sample number regarding sex, site, age, and condition; File S2: ASVs identified as contaminants and removed; File S3: ASVs identified as contaminants and removed; File S4: Statistical analyses for diversity measurements.
