# Supplementary material for: Characterization of the Blood Bacterial Microbiota in Lowland Tapirs (Tapirus terrestris), a Vulnerable Species in Brazil
Source: Microorganisms. 2024 Nov 8;12(11):2270. doi: 10.3390/microorganisms12112270 (PMC11596849; doi:10.3390/microorganisms12112270)
Supplement: Supplementary file 1 [file microorganisms-12-02270-s001.zip › supplementary file S4.pdf]

Table S1. Kruskal-Wallis statistical results comparing the Shannon index for alpha diversity between blood samples obtained from living and road-killed tapirs (values were considered significant when *p*-value < 0.05).

**Group 1**	**Group 2**	**H**	***p*-value**	**q-value**
Road-killed (n=3)	Living (n=63)	8.462687	0.003625	0.003625

Table S2. Statistical analysis comparing the distances calculated using Pairwise PERMANOVA test between bacterial communities found in blood samples obtained from living and road-killed tapirs.

**Group 1**	**Group 2**	**Sample size**	**Permutations**	**pseudo-F**	***p*-value**	**q-value**
Road-killed	Living	66	999	49.581599	0.001	0.001

Table S3. Statistical analysis comparing the distances calculated using Pairwise PERMANOVA test between bacterial communities found in blood samples obtained from tapirs from Cerrado and Pantanal biomes.

**Group 1**	**Group 2**	**Sample size**	**Permutations**	**pseudo-F**	***p*-value**	**q-value**
Cerrado	Pantanal	66	999	2.982857	0.033	0.033

Table S4. Statistical analysis comparing the distances calculated using Pairwise PERMANOVA test between bacterial communities found in blood samples obtained from adult, sub-adult and juvenile tapirs.

**Group 1**	**Group 2**	**Sample size**	**Permutations**	**pseudo-F**	***p*-value**	**q-value**
Adult	Juvenile	39	999	1.191616	0.328	0.492

	Sub-Adult	60	999	4.413034	0.007	0.021
Juvenile	Sub-Adult	33	999	0.219951	0.794	0.794

Table S5. Statistical analysis comparing the distances calculated using Pairwise PERMANOVA test between bacterial communities found in blood samples obtained from adults, sub-adults and juveniles in living animals' samples.

Group 1	Group 2	Sample size	Permutations	pseudo-F	*p*-value	q-value
Adult	Juvenile	38	999	1.090382	0.191	0.2865
	Sub-Adult	58	999	6.611545	0.001	0.0030
Juvenile	Sub-Adult	32	999	0.260229	0.735	0.7350
