# Supplementary material for: Characterization of the Blood Bacterial Microbiota in Lowland Tapirs (Tapirus terrestris), a Vulnerable Species in Brazil
Source: Microorganisms. 2024 Nov 8;12(11):2270. doi: 10.3390/microorganisms12112270 (PMC11596849; doi:10.3390/microorganisms12112270)
Supplement: Supplementary file 1 [file microorganisms-12-02270-s001.zip › supplementaryfile S1.pdf]

number	sex	age	site	dead_or_living
numeric	categorical	categorical	categorical	categorical
1	Male	adult	Cerrado	living
2	Male	subadult	Cerrado	living
3	Male	adult	Cerrado	living
4	Female	adult	Cerrado	living
5	Female	adult	Cerrado	living
6	Female	adult	Cerrado	living
7	Female	subadult	Cerrado	living
8	Male	adult	Cerrado	living
9	Female	juvenile	Cerrado	living
10	Female	adult	Cerrado	living
11	Female	adult	Cerrado	living
13	Male	subadult	Cerrado	living
14	Male	juvenile	Cerrado	living
17	Female	adult	Cerrado	living
18	Female	adult	Cerrado	living
19	Female	adult	Cerrado	living
20	Male	adult	Cerrado	living
21	Female	subadult	Cerrado	living
22	Female	subadult	Cerrado	living
23	Female	adult	Cerrado	living
24	Male	subadult	Cerrado	living
26	Male	adult	Cerrado	living
27	Female	adult	Cerrado	living
28	Female	juvenile	Cerrado	living
29	Male	subadult	Cerrado	living
30	Female	adult	Cerrado	living
31	Male	adult	Cerrado	living
32	Male	subadult	Cerrado	living
33	Male	subadult	Cerrado	living
35	Female	subadult	Pantanal	living
36	Male	juvenile	Pantanal	living
37	Male	adult	Pantanal	living
38	Female	adult	Pantanal	living
40	Male	subadult	Pantanal	living
42	Male	adult	Pantanal	living
43	Female	adult	Pantanal	living
44	Male	subadult	Pantanal	living
45	Male	subadult	Pantanal	living
48	Male	subadult	Pantanal	living
53	Male	subadult	Pantanal	living
56	Male	subadult	Pantanal	living
57	Female	adult	Pantanal	living
60	Male	subadult	Pantanal	living
62	Male	juvenile	Pantanal	living
64	Male	juvenile	Pantanal	living
66	Female	juvenile	Pantanal	living
68	Male	adult	Pantanal	living
70	Male	adult	Pantanal	living
72	Male	subadult	Pantanal	living
74	Male	adult	Pantanal	living
77	Male	adult	Pantanal	living
78	Female	subadult	Pantanal	living
80	Male	subadult	Pantanal	living
81	Male	adult	Pantanal	living
83	Male	subadult	Pantanal	living
85	Female	adult	Pantanal	living
86	Female	adult	Pantanal	living
87	Female	adult	Pantanal	living
88	Male	adult	Pantanal	living
91	Female	subadult	Pantanal	living
93	Male	juvenile	Pantanal	living
95	Female	adult	Pantanal	living
96	Female	adult	Pantanal	living
98	Female	adult	Cerrado	living
100	Female	adult	Cerrado	living
101	Female	adult	Cerrado	living
103	Male	adult	Pantanal	living
105	Female	adult	Pantanal	living
106	Male	subadult	Pantanal	living
108	Female	subadult	Pantanal	living
109	Male	subadult	Pantanal	living
110	Male	subadult	Pantanal	living
111	Male	subadult	Pantanal	living
112	Male	subadult	Pantanal	living
113	Male	subadult	Pantanal	living
114	Female	subadult	Pantanal	living
115	Female	subadult	Pantanal	living
116	Male	adult	Pantanal	living
118	Female	subadult	Cerrado	dead
119	Female	juvenile	Cerrado	dead
121	Male	adult	Cerrado	dead
122	Male	adult	Cerrado	dead
123	Male	adult	Cerrado	dead
124	Female	adult	Cerrado	dead
125	Male	subadult	Cerrado	dead
126	Female	adult	Cerrado	living
